# Supplementary material for: HIVprotI: an integrated web based platform for prediction and design of HIV proteins inhibitors
Source: J Cheminform. 2018 Mar 9;10:12. doi: 10.1186/s13321-018-0266-y (PMC5845081; doi:10.1186/s13321-018-0266-y)
Supplement: Supplementary file 2 — Additional file 2. Source code of HIVProtI web server. [file 13321_2018_266_MOESM2_ESM.zip › HIVprotI_Source-code/hivprotI/weka-3-6-6/doc/weka/filters/MultiFilter.html]

MultiFilter


---


|  |  |  |
| --- | --- | --- |
| **Overview** \| **Package** \| **Class** \| **Tree** \| **Deprecated** \| **Index** \| **Help** \| **Weka's home** | |  |
| **PREV CLASS**   **NEXT CLASS** | **FRAMES**    **NO FRAMES**     **All Classes** |  |
| SUMMARY: NESTED \| FIELD \| CONSTR \| METHOD | DETAIL: FIELD \| CONSTR \| METHOD |  |


---


## weka.filters Class MultiFilter

```
java.lang.Object
  weka.filters.Filter
      weka.filters.SimpleFilter
          weka.filters.SimpleStreamFilter
              weka.filters.MultiFilter
```

**All Implemented Interfaces:**: java.io.Serializable, CapabilitiesHandler, OptionHandler, RevisionHandler, StreamableFilter

---

``` public class MultiFilter extends SimpleStreamFilter ```

Applies several filters successively. In case all supplied filters are StreamableFilters, it will act as a streamable one, too.


Valid options are:

```
 -D
  Turns on output of debugging information.
```

```
 -F <classname [options]>
  A filter to apply (can be specified multiple times).
```

**Version:**
:   $Revision: 6839 $

**Author:**
:   FracPete (fracpete at waikato dot ac dot nz)

**See Also:**: `StreamableFilter`, Serialized Form

---

**Constructor Summary**

`MultiFilter()`


| **Method Summary** | |
| --- | --- |
| `boolean` | `batchFinished()`             Signify that this batch of input to the filter is finished. |
| `java.lang.String` | `filtersTipText()`             Returns the tip text for this property |
| `Capabilities` | `getCapabilities()`             Returns the Capabilities of this filter. |
| `Filter` | `getFilter(int index)`             Gets a single filter from the set of available filters. |
| `Filter[]` | `getFilters()`             Gets the list of possible filters to choose from. |
| `java.lang.String[]` | `getOptions()`             Gets the current settings of the filter. |
| `java.lang.String` | `getRevision()`             Returns the revision string. |
| `java.lang.String` | `globalInfo()`             Returns a string describing this filter |
| `boolean` | `isStreamableFilter()`             tests whether all the enclosed filters are streamable |
| `java.util.Enumeration` | `listOptions()`             Returns an enumeration describing the available options. |
| `static void` | `main(java.lang.String[] args)`             Main method for executing this class. |
| `void` | `setFilters(Filter[] filters)`             Sets the list of possible filters to choose from. |
| `void` | `setOptions(java.lang.String[] options)`             Parses a list of options for this object. |

**Methods inherited from class weka.filters.SimpleStreamFilter**

`input`

**Methods inherited from class weka.filters.SimpleFilter**

`debugTipText, getDebug, setDebug, setInputFormat`

**Methods inherited from class weka.filters.Filter**

`batchFilterFile, filterFile, getCapabilities, getOutputFormat, isFirstBatchDone, isNewBatch, isOutputFormatDefined, makeCopies, makeCopy, numPendingOutput, output, outputPeek, toString, useFilter, wekaStaticWrapper`

**Methods inherited from class java.lang.Object**

`equals, getClass, hashCode, notify, notifyAll, wait, wait, wait`

**Constructor Detail**

### MultiFilter

```
public MultiFilter()
```


**Method Detail**

### globalInfo

```
public java.lang.String globalInfo()
```

:   Returns a string describing this filter

    :   **Specified by:**: `globalInfo` in class `SimpleFilter`
    :   **Returns:**: a description of the filter suitable for displaying in the explorer/experimenter gui

---


### listOptions

```
public java.util.Enumeration listOptions()
```

:   Returns an enumeration describing the available options.

    :   **Specified by:**: `listOptions` in interface `OptionHandler` **Overrides:**: `listOptions` in class `SimpleFilter`
    :   **Returns:**: an enumeration of all the available options.

---


### setOptions

```
public void setOptions(java.lang.String[] options)
                throws java.lang.Exception
```

:   Parses a list of options for this object.


    Valid options are:

    ```
     -D
      Turns on output of debugging information.
    ```

    ```
     -F <classname [options]>
      A filter to apply (can be specified multiple times).
    ```

    :   **Specified by:**: `setOptions` in interface `OptionHandler` **Overrides:**: `setOptions` in class `SimpleFilter`
    :   **Parameters:**: `options` - the list of options as an array of strings **Throws:**: `java.lang.Exception` - if an option is not supported **See Also:**: `SimpleFilter.reset()`

---


### getOptions

```
public java.lang.String[] getOptions()
```

:   Gets the current settings of the filter.

    :   **Specified by:**: `getOptions` in interface `OptionHandler` **Overrides:**: `getOptions` in class `SimpleFilter`
    :   **Returns:**: an array of strings suitable for passing to setOptions

---


### getCapabilities

```
public Capabilities getCapabilities()
```

:   Returns the Capabilities of this filter.

    :   **Specified by:**: `getCapabilities` in interface `CapabilitiesHandler` **Overrides:**: `getCapabilities` in class `Filter`
    :   **Returns:**: the capabilities of this object **See Also:**: `Capabilities`

---


### setFilters

```
public void setFilters(Filter[] filters)
```

:   Sets the list of possible filters to choose from.
    Also resets the state of the filter (this reset doesn't affect the
    options).

    :   **Parameters:**: `filters` - an array of filters with all options set. **See Also:**: `reset()`

---


### getFilters

```
public Filter[] getFilters()
```

:   Gets the list of possible filters to choose from.

    :   **Returns:**: the array of Filters

---


### filtersTipText

```
public java.lang.String filtersTipText()
```

:   Returns the tip text for this property

    :   **Returns:**: tip text for this property suitable for displaying in the explorer/experimenter gui

---


### getFilter

```
public Filter getFilter(int index)
```

:   Gets a single filter from the set of available filters.

    :   **Parameters:**: `index` - the index of the filter wanted **Returns:**: the Filter

---


### isStreamableFilter

```
public boolean isStreamableFilter()
```

:   tests whether all the enclosed filters are streamable

    :   **Returns:**: true if all the enclosed filters are streamable

---


### batchFinished

```
public boolean batchFinished()
                      throws java.lang.Exception
```

:   Signify that this batch of input to the filter is finished. If
    the filter requires all instances prior to filtering, output()
    may now be called to retrieve the filtered instances. Any
    subsequent instances filtered should be filtered based on setting
    obtained from the first batch (unless the setInputFormat has been
    re-assigned or new options have been set).

    :   **Overrides:**: `batchFinished` in class `SimpleStreamFilter`
    :   **Returns:**: true if there are instances pending output **Throws:**: `java.lang.IllegalStateException` - if no input format has been set.: `java.lang.NullPointerException` - if no input structure has been defined,: `java.lang.Exception` - if there was a problem finishing the batch.

---


### getRevision

```
public java.lang.String getRevision()
```

:   Returns the revision string.

    :   **Specified by:**: `getRevision` in interface `RevisionHandler` **Overrides:**: `getRevision` in class `Filter`
    :   **Returns:**: the revision

---


### main

```
public static void main(java.lang.String[] args)
```

:   Main method for executing this class.

    :   **Parameters:**: `args` - should contain arguments for the filter: use -h for help


---


|  |  |  |
| --- | --- | --- |
| **Overview** \| **Package** \| **Class** \| **Tree** \| **Deprecated** \| **Index** \| **Help** \| **Weka's home** | |  |
| **PREV CLASS**   **NEXT CLASS** | **FRAMES**    **NO FRAMES**     **All Classes** |  |
| SUMMARY: NESTED \| FIELD \| CONSTR \| METHOD | DETAIL: FIELD \| CONSTR \| METHOD |  |


---
